# Monitoring of Non-communicable Diseases in a Primary Healthcare Setting in India: A Quality Improvement Initiative

**DOI:** 10.7759/cureus.38132

**Published:** 2023-04-25

**Authors:** Ankit Chandra, Ravneet Kaur, Mohan Bairwa, Sanjay Rai, Baridalyne Nongkynrih

**Affiliations:** 1 Centre for Community Medicine, All India Institute of Medical Sciences, New Delhi, IND

**Keywords:** qi study, hypertension, diabetes, control rate, ncd

## Abstract

Background

The majority of patients with non-communicable diseases (NCDs) seek care in a primary healthcare setting. There is a lack of effective monitoring of patients with NCD, which leads to poor disease control and an increase in morbidity and mortality. We wanted to explore the feasibility of maintaining patient health record and utilising it for disease monitoring in a primary healthcare setting. Therefore, we aimed to increase the availability of patient health records from 0% to 100% using the principles of quality improvement (QI) among patients with hypertension and/or diabetes within six weeks and to use these records for assessing the disease control status of patients through cohort monitoring approach.

Methods

This QI initiative was conducted at an urban health centre (UHC) located at Dakshinpuri, New Delhi. We specifically focused on two major NCDs: diabetes and hypertension. We formed a QI team and identified the gaps using fishbone analysis and a process flow diagram. We used the model for improvement and the Plan-Do-Study-Act (PDSA) framework. We conducted repeated rapid PDSA cycles for the designed intervention and monitored the change every week using a run chart. The data from the patient health record were entered into Microsoft Excel (Microsoft^®^ Corp., Redmond, WA) using Google Forms (Google, Inc., Mountain View, CA) and Epicollect5 (Oxford Big Data Institute, Oxford, England). We used the cohort monitoring approach of the India Hypertension Control Initiative to assess the quarterly control rate for hypertension and diabetes at the UHC.

Results

The root cause analysis revealed that the lack of a policy for keeping patient records and the lack of perceived need in the past were the primary reasons behind the absence of NCD health records. In brainstorming sessions with the QI team, we designed a paper-based patient health record system involving unique identity (ID) generation, an index register, an NCD record file and an NCD passbook (Dhirghayu card) for each patient. We reorientated the process of patient flow and devised a mechanism for record-keeping at the UHC. This initiative increased the availability of patient health records from 0% to 100% in the initial three weeks. The system of maintaining patient health records was well received by the patients and was better utilised by treating physicians for NCD management. After the intervention, we were able to use the data from the NCD file to assess the quarterly control rates of the patients with hypertension and/or diabetes.

Conclusion

Our study showed that patients’ health records can be generated and maintained in a primary healthcare setting by using the principles of quality improvement. These records can be utilised for the disease monitoring of patients with hypertension and/or diabetes, which can lead to better disease control. The sustainability of this initiative and the performance of the health facility can be assessed in future studies using annual control rates.

## Introduction

Non-communicable diseases (NCDs) account for 60% of all deaths in India [1]. To tackle this burden, India launched the National Programme for Prevention and Control of Cancer, Diabetes, Cardiovascular Diseases and Stroke (NPCDCS) in 2010. Under the NPCDCS programme, curative and preventive services are being provided [2]. Despite more than a decade of implementation, the quality of care and services for NCDs remains suboptimum. Most patients with NCDs seek care at primary health centres, which are tasked with a vital role in managing NCDs. However, a national survey has revealed the poor quality of care for NCDs at primary health centres in India, including a lack of availability of essential technologies and medicines, a shortage of nurse-midwives and health assistants and inadequate training under NPCDCS [3]. One of the objectives of the NPCDCS is to develop the database of NCDs through surveillance and to monitor NCD morbidity and risk factors [2]. However, there is a lack of reliable and adequate data from the health facility level [4]. Health facility-based data are essential for estimating the requirement of drugs and supplies, which ultimately affects the quality of healthcare services. Patients with NCDs need regular monitoring, refill of medications and appropriate management. There is a lack of effective monitoring in primary care settings. The lack of NCD monitoring affects the control rate of the disease, subsequently leading to increased morbidity and mortality [5].

Quality improvement (QI) is a management approach that health workers can use to reorganise patient care at their level to ensure that patients receive good-quality healthcare [6]. Quality improvement initiatives are essential for identifying and addressing gaps in care delivery. In our primary care facility, we found a lack of availability of patient health records. The patient health record is a lifelong record of health-related information of an individual [7]. It consists of all the past and current medical history, examination findings, laboratory reports and past and current treatment details. There is a need for a quality improvement initiative to address the gaps in the monitoring of patients with NCDs. Through this study, we attempted to strengthen the NCD care services in a primary care setting using the principles of QI, which is a key strategy for achieving universal health coverage and improving health outcomes. We wanted to explore the feasibility of maintaining patient health record and utilising it for disease monitoring in a primary healthcare setting. Therefore, we aimed to increase the availability of patient health records from 0% to 100% among patients with hypertension and/or diabetes seeking care at the primary healthcare setting within six weeks. Additionally, we aimed to utilise these records for assessing the disease control status of patients through cohort monitoring.

## Materials and methods

**Figure 1:**
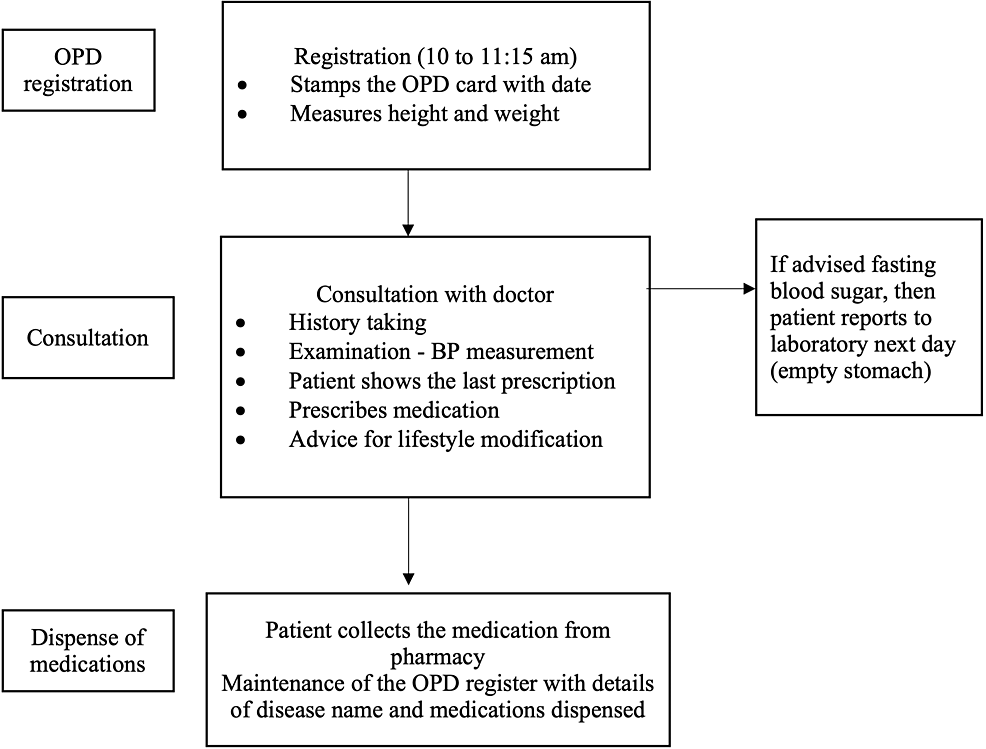
Process flow diagram showing the patient pathway at an urban health centre at Dakshinpuri, New Delhi OPD, outpatient department; BP, blood pressure

This QI initiative was conducted at a primary healthcare setting, which is an urban health centre (UHC) located at Dakshinpuri, New Delhi, India. The UHC caters to a resettlement colony consisting of 5,561 households and a population of up to 38,000. This UHC was adopted as a part of the urban health programme in 2013. It provides primary healthcare for NCDs and has around 4-5 resident doctors for consultation. The UHC is operational five days a week from 10 am to 12 pm. In this study, we specifically focused on two major NCDs: diabetes and hypertension, which collectively constitute a significant portion of all NCDs. At our primary health centre, these conditions account for more than 60% of all consultations annually. The average number of consultations for patients with either of these conditions is 16 per day. We formed a QI team, which constituted a medical officer (team leader for training, implementing and supervising), a medical social service officer (for supportive supervision), a public health nurse and a registration counter clerk for the implementation of the intervention. We identified the problem as the lack of availability of the patient health record, which is essential for the monitoring of patients with NCD and from the perspective of health system management. To identify the root cause of the problem, we first conducted a baseline assessment and mapped the existing process using a process flow diagram (Figure 1). We then used fishbone analysis to identify the root cause of the problem.

We utilised the model for improvement and the Plan-Do-Study-Act (PDSA) framework for this QI initiative. The change was defined as the availability of the patient health record among patients with diabetes and/or hypertension in an UHC at Dakshinpuri, New Delhi. We used a run chart to monitor the change, and it was monitored every week by the medical officer and medical social service officer. We included UHC staff and adult patients (age of ≥18 years) with NCDs (diabetes/hypertension) for this study and used the PDSA framework for testing the intervention. We collected data from 30 consecutive patients in the baseline assessment, and 30 patients were selected every week after the intervention for rapid multiple PDSA cycles. The data were collected using a semi-structured questionnaire, prescription audit and record review by the QI team. We used the cohort monitoring approach of the India Hypertension Control Initiative to assess the quarterly control rate for hypertension and/or diabetes at the UHC [8]. Disease status for hypertensive patients was considered as ‘controlled’ if their blood pressure was <140/90 in the most recent visit in the assessment quarter [9]. The disease status of patients with diabetes was considered ‘controlled’ if the latest fasting blood sugar was ≤126 mg/dl or the haemoglobin A1c was ≤7% in the assessment quarter [10]. The implementation of the intervention and PDSA cycles were conducted in September 2021. Patients registered between October and December 2021 were assessed for control during January-March 2022 as the first quarter. Patients registered between January and March 2022 were assessed for control during April-June 2022 as the second quarter. Data from the NCD record file were quarterly entered into the Microsoft Excel sheet (Microsoft® Corp., Redmond, WA) using Google Forms (Google, Inc., Mountain View, CA) and Epicollect5 (Oxford Big Data Institute, Oxford, England). A descriptive analysis was done using R software (R Foundation for Statistical Computing, Vienna, Austria). Ethical approval was obtained from the Institute Ethics Committee of All India Institute of Medical Sciences (AIIMS), New Delhi, before starting the study (reference number: IEC-34/14.01.2022).

## Results

**Figure 2:**
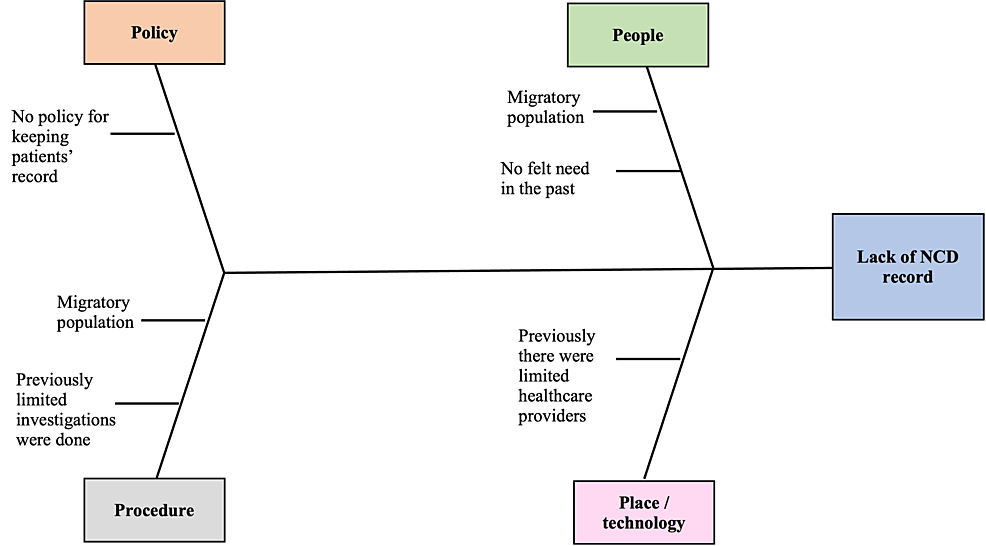
Fishbone diagram illustrating the root cause analysis conducted to identify the reasons behind the lack of NCD record availability NCD: non-communicable disease

The QI team had identified the gap in the service delivery to be the absence of availability of the patient health records, leading to irrational indenting of the medications, and poor patient monitoring (as all the patients were not bringing their previous prescription cards or reports). Additionally, frequent changes in treating physicians made it challenging for other doctors to adjust the dosages of medications. The root cause analysis showed that the primary reasons behind the lack of NCD health records were the absence of a policy for keeping patient records and the lack of perceived need in the past (Figure 2).

**Figure 3:**
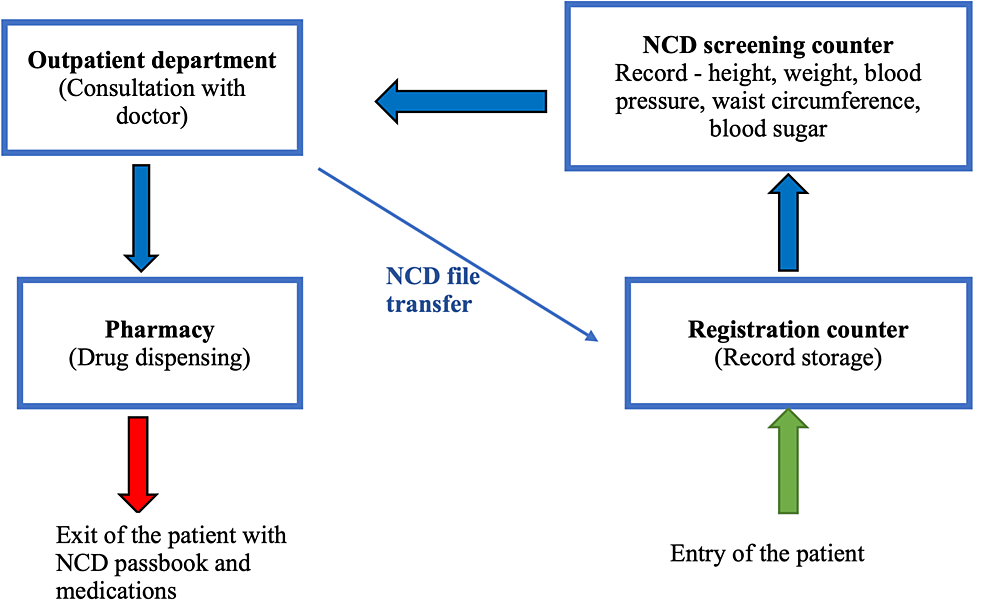
Reorientation of the patient process flow at an urban health centre at Dakshinpuri, New Delhi NCD: non-communicable disease

To address the issue of the lack of NCD health records of the patients, we developed a system to generate and maintain paper-based patient health records [11]. This consisted of unique identity (ID) generation, index register, NCD record file and NCD passbook for each patient. The index register contained the list of patients with NCD and their unique ID codes, which was helpful in retrieving a patient’s ID in case the patient forgot it. The NCD record file, kept at the UHC, served as the patient health record, while the NCD passbook contained the same information that patients brought with them on each visit to the UHC. We named the NCD passbook ‘Dhirghayu card’ in the local language to increase its acceptance in the community. These two records (NCD file and passbook) contained the details of previous prescriptions, examination findings, investigation reports and previous visits to the UHC. We also reorientated the process of patient flow (Figure 3) and devised a mechanism for record-keeping at the UHC.

**Figure 4:**
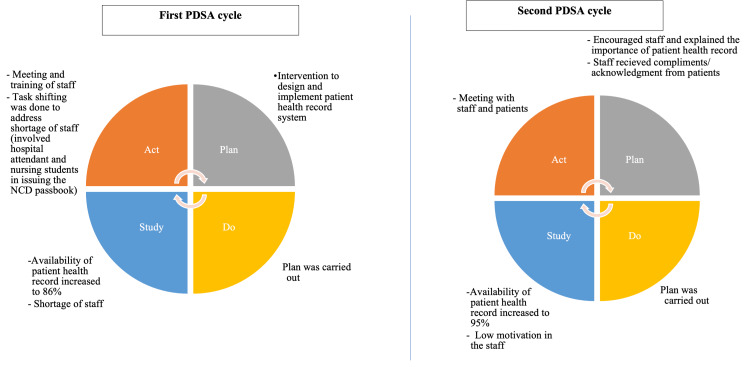
Plan-Do-Study-Act (PDSA) cycles carried out to test the change for increasing the availability of the patient health record among patients with non-communicable diseases (NCDs) in an urban health centre at Dakshinpuri, New Delhi

**Figure 5:**
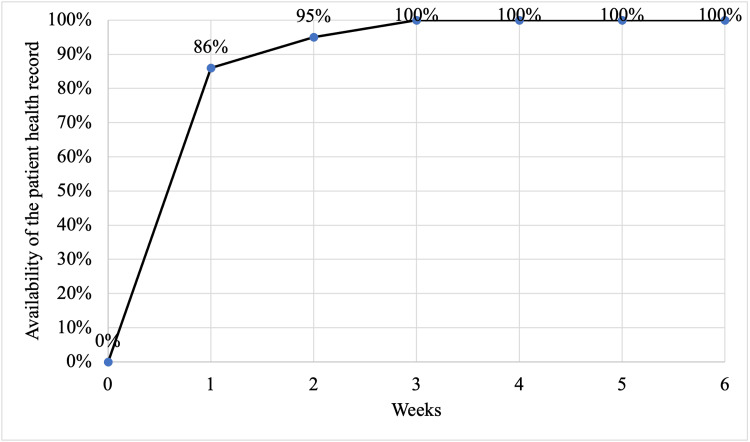
Run chart showing the availability of the patient health record among patients with diabetes and/or hypertension in an urban health centre at Dakshinpuri, New Delhi

The change was tested using PDSA cycles (Figure 4). In the first PDSA cycle, we faced the problem of a shortage of manpower for this, and the staff felt overburdened. We overcame it by task shifting and after receiving positive feedback from the patients. We trained the hospital attendant in issuing the NCD passbook and NCD file creation with the help of students posted at the centre for their training. In the second PDSA cycle, we achieved a significant proportion of patients with health records. During this process, we felt a low motivation among the staff, which was managed well with the appreciation and acknowledgment of their crucial work done for patient care. This system of maintaining patient health records was well appreciated by the patients as they did not have to carry a load of papers/files. A few treating physicians did mention that it increased the workload due to the duplication of the patient data in the NCD file and NCD passbook. However, it was greatly appreciated as these records were better utilised by the treating physician for their decision-making regarding NCD management. After the implementation of this intervention, the availability of patient health records increased from 0% to 100% in the initial three weeks (Figure 5).

**Figure 6:**
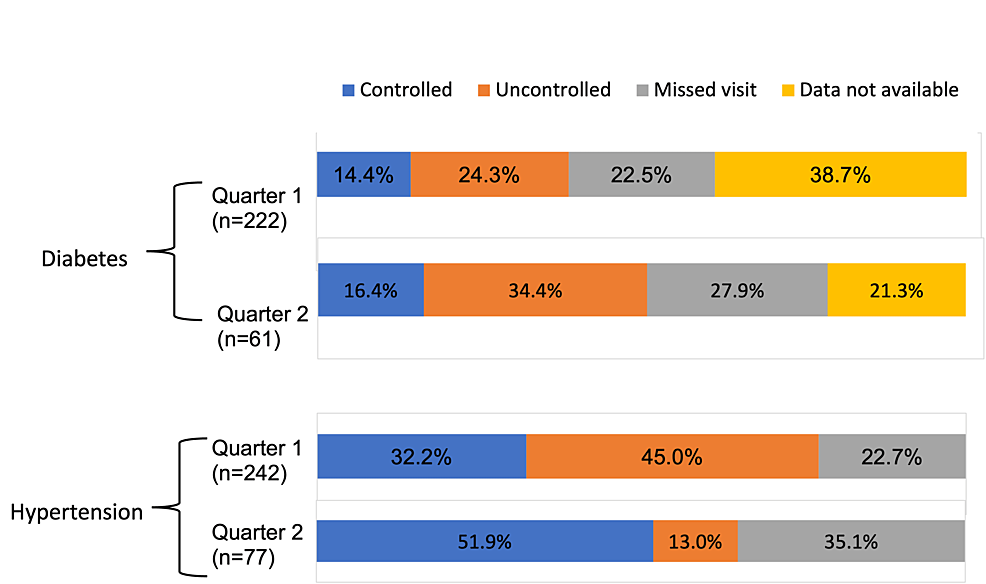
Quarterly outcome of patients with diabetes and hypertension registered at an urban health centre at Dakshinpuri, New Delhi

In the first quarter, a total of 343 patients were registered (old/new) at an urban health centre at Dakshinpuri, New Delhi. Out of these, 121 patients had only hypertension, 101 patients had only diabetes and 121 had both diabetes and hypertension. In the second quarter, a total of 115 patients (old/new) were registered, with 54 patients having only hypertension, 38 patients having only diabetes and 23 patients having both diabetes and hypertension. Among the patients with hypertension, the proportion of patients with controlled blood pressure was 32.2% in the first quarter and 51.9% in the second quarter. Among the patients with diabetes, the proportion of patients with controlled blood sugar was 14.4% in the first quarter and 16.4% in the second quarter (Figure 6).

## Discussion

This paper describes a quality improvement initiative carried out in an urban health centre at Dakshinpuri, New Delhi, to address the lack of availability of patient health records for patients with non-communicable diseases (NCDs). We were able to increase the availability of patient health records from 0% to 100% among patients with hypertension and/or diabetes seeking care in primary healthcare setting within three weeks using the principles of quality improvement and tools such as fishbone analysis, process flow diagram, run chart and PDSA cycle [6]. In this QI initiative, our intervention included the generation of unique IDs for patients and the generation and maintenance of patient health records in a primary care setting. These records were used for disease monitoring through a cohort monitoring approach, and generating such health records/data at the primary care level will help in improving the quality of patient care services for NCDs.

Similar to the other studies done in the past [12,13], we faced challenges such as a shortage of staff and low work motivation during the implementation. It is a usual thing, as people tend to avoid any change in the systems. In this study, we only analysed data from two quarters, which may not provide a comprehensive view of patient profiles and control status. To better understand the effectiveness of the intervention, it is recommended to use facility-based or community-based annual indicators, which will provide a more comprehensive overview of the patients’ control status. Additionally, it is important to evaluate the sustainability of the intervention over a few years and use data on control status to assess the performance of the health facility based on annual indicators. The quarterly indicators used in this study did provide vital information on the uncontrolled status and missed visits of patients. And among the diabetics, the majority of the patients did not have the report of their glycaemic status or missed the follow-up visits. The missed visits among the patients could be high due to the migration among the catered population as the proportion of patients is quite similar to the out-migration rate of our study area. Further interventions can be considered to increase the follow-up visits and blood sugar testing at the primary care level. This intervention was done at a public health facility; further study can be done involving private health facilities. A study done among private practitioners in Pune had shown a positive response and cooperation towards NCD surveillance [14].

To our knowledge, this is the first study from India that applied the principles of quality improvement in strengthening NCD services in the primary care setting. For the implementation of this intervention, we did have adequate skilled manpower and resources as our UHC is affiliated with an academic institution. However, we do believe that this can be replicated in other primary care settings, as this involves simple techniques that will help in improving NCD management. This intervention was well accepted by the staff and study population. All patients who attended the outpatient department (OPD) had their blood pressure data recorded and available for analysis. A periodic assessment of this intervention will be in place to ensure sustainability.

The limitation of this study was that it was conducted in an urban setting, in a single health centre, and included monitoring of only selected NCDs. It does not include other NCDs such as cancers, mental health ailments, cardiac illness, stroke, chronic obstructive pulmonary disease, asthma and hypothyroidism. The implementation of a similar intervention in a rural setting might face different challenges due to the variation in study participants and manpower. Since this was a paper-based health record system, our staff did face the challenge of increased paperwork and maintenance and a shortage of space at the UHC. Further interventions can be directed towards electronic health records, which will reduce the paperwork and will help in real-time monitoring. The digitalisation of these health records and integration at a national level will be a key step towards NCD management. The study’s limitations also include a small sample size and a control analysis of two quarters only.

## Conclusions

Patients’ health records can be maintained in a primary healthcare setting by using the principles of quality improvement. They can be utilised for the monitoring of patients with NCDs. India is moving towards digitalising and, under the digital health mission initiative, has introduced the system of a unique health ID for all its citizens. This is the first step towards creating safer and more efficient digital health records and will go a long way in improving the patient monitoring system.
