# Correction: Monitoring of Non-communicable Diseases in a Primary Healthcare Setting in India: A Quality Improvement Initiative

**DOI:** 10.7759/cureus.c112

**Published:** 2023-04-28

**Authors:** Ankit Chandra, Ravneet Kaur, Mohan Bairwa, Sanjay Rai, Baridalyne Nongkynrih

**Affiliations:** 1 Centre for Community Medicine, All India Institute of Medical Sciences, New Delhi, IND

This article has been corrected to replace Figure 6 as the original version incorrectly depicted the Diabetes Quarter 1 control rate as 51.9% instead of the correct  14.4%. The authors and editorial staff regret this error was not identified prior to publication.

